# Pulmonary metastasectomy and repeat metastasectomy for colorectal pulmonary metastases: outcomes from the Dutch Lung Cancer Audit for Surgery

**DOI:** 10.1093/bjsopen/zrad009

**Published:** 2023-05-05

**Authors:** Martijn van Dorp, Nienke Wolfhagen, Bart Torensma, Chris Dickhoff, Geert Kazemier, David J Heineman, Wilhelmina H Schreurs

**Affiliations:** Department of Cardiothoracic Surgery, Amsterdam University Medical Centre, Amsterdam, The Netherlands; Dutch Institute for Clinical Auditing, Leiden, The Netherlands; Department of Anaesthesiology, Leiden University Medical Centre, Leiden, The Netherlands; Department of Cardiothoracic Surgery, Amsterdam University Medical Centre, Amsterdam, The Netherlands; Department of Surgery, Amsterdam University Medical Centre, Amsterdam, The Netherlands; Department of Cardiothoracic Surgery, Amsterdam University Medical Centre, Amsterdam, The Netherlands; Department of Surgery, Northwest Clinics, Alkmaar, The Netherlands

## Abstract

**Background:**

Surgical resection of recurrent pulmonary metastases in patients with colorectal cancer is an established treatment option; however, the evidence for repeat resection is limited. The aim of this study was to analyse long-term outcomes from the Dutch Lung Cancer Audit for Surgery.

**Methods:**

Data from the mandatory Dutch Lung Cancer Audit for Surgery were used to analyse all patients after metastasectomy or repeat metastasectomy for colorectal pulmonary metastases from January 2012 to December 2019 in the Netherlands. Kaplan–Meier survival analysis was performed to determine the difference in survival. Multivariable Cox regression analyses were performed to identify predictors of survival.

**Results:**

A total of 1237 patients met the inclusion criteria, of which 127 patients underwent repeat metastasectomy. Five-year overall survival was 53 per cent after pulmonary metastasectomy for colorectal pulmonary metastases and 52 per cent after repeat metastasectomy (*P* = 0.852). The median follow-up was 42 (range 0–285) months. More patients experienced postoperative complications after repeat metastasectomy compared with the first metastasectomy (18.1 per cent *versus* 11.6 per cent respectively; *P* = 0.033). Eastern Cooperative Oncology Group performance status greater than or equal to 1 (HR 1.33, 95 per cent c.i. 1.08 to 1.65; *P* = 0.008), multiple metastases (HR 1.30, 95 per cent c.i. 1.01 to 1.67; *P* = 0.038), and bilateral metastases (HR 1.50, 95 per cent c.i. 1.01 to 2.22; *P* = 0.045) were prognostic factors on multivariable analysis for pulmonary metastasectomy. Diffusing capacity of the lungs for carbon monoxide less than 80 per cent (HR 1.04, 95 per cent c.i. 1.01 to 1.06; *P* = 0.004) was the only prognostic factor on multivariable analysis for repeat metastasectomy.

**Conclusion:**

This study demonstrates that patients with colorectal pulmonary metastases have comparable median and 5-year overall survival rates after primary or recurrent pulmonary metastasectomy. However, repeat metastasectomy has a higher risk of postoperative complications.

## Teaser

Analysis of the Dutch national registry demonstrates that patients with colorectal pulmonary metastases have comparable median and 5-year overall survival rates after primary or recurrent pulmonary metastasectomy. Repeat metastasectomy has a higher risk of postoperative complications. The number of metastases, bilateral metastases, impaired diffusing capacity of the lungs for carbon monoxide, and Eastern Cooperative Oncology Group performance status predict poor survival after metastasectomy.

## Introduction

Pulmonary metastasectomy for colorectal pulmonary metastases (CRPM) has a reported 5-year overall survival rate of 44 per cent (95 per cent c.i. 40.5 to 48.7 per cent)^1^. Despite receiving treatment with curative intent, 43–58 per cent of patients experience pulmonary disease recurrence^2-8^, with a median time to pulmonary recurrence of 13–19 months^4,5,7,9,10^. Although supporting evidence in favour of repeat metastasectomy is limited, selected studies have demonstrated 5-year overall survival rates after repeat metastasectomy varying from 51 to 79 per cent^9-18^. However, reported repeat metastasectomy rates vary between 10 and 26 per cent^2,9-11,14,16-19^. Repeat metastasectomy may increase the risk of postoperative morbidity rate compared with the first metastasectomy due to impaired residual lung capacity and pleural adhesions^20^. Alternatively to repeat metastasectomy, stereotactic radiotherapy can be a valid treatment option; however, long-term follow-up suggests an increased risk of local recurrence^21,22^. Extensive literature is available on prognostic factors for survival after pulmonary metastasectomy in colorectal cancer patients^1,23,24^. However, most of the known prognosticators for the first pulmonary metastasectomy have not been proven as prognostic factors for survival for repeat metastasectomy^9,10,12,14,16^.

The aim of this study was to analyse the long-term survival, postoperative morbidity rate, and prognosticators of patients registered in the Dutch Lung Cancer Audit for Surgery (DLCA-S) who underwent pulmonary metastasectomy and repeat metastasectomy for CRPM.

## Methods

Data were retrieved from the DLCA-S database, after approval of the Privacy Review Board of the DLCA-S (M21101276—DLCAS202007), in accordance with the Dutch Personal Data Protection Act. The DLCA-S is a mandatory prospective nationwide quality registry run by the Dutch Institute for Clinical Auditing (DICA) that has collected data from all thoracic surgery units in the Netherlands since 2012^25^.

### Patients

Patients were selected for analysis if they underwent metastasectomy of CRPM between 1 January 2012 and 31 December 2019. The citizen service numbers of these patients were linked to the Vektis database, containing data from all Dutch healthcare-insured citizens. They provided long-term survival data of all patients with an available citizen service number. Healthcare insurance is compulsory for Dutch citizens and 99 per cent of Dutch inhabitants have private healthcare insurance^26^. Retrieval of survival status was completed until 23 November 2021. Eligibility of analysis was based on minimal data requirements (including age, sex, date of surgery, type of parenchymal resection, location of the metastasis, and postoperative mortality rate) from the DLCA-S database and on pathologically proven colorectal cancer histology.

### Outcomes

Patient characteristics included age, sex, Eastern Cooperative Oncology Group (ECOG) performance status, ASA classification, Charlson co-morbidity index (CCI), pulmonary function testing using the forced expiratory volume in 1 s (FEV1) in percentage of predicted and diffusing capacity of the lungs for carbon monoxide (DLCO) in percentage of predicted, and cardiopulmonary exercise testing using maximal oxygen consumption (VO_2_ max) in ml/kg/min. Treatment characteristics included the extent of resection, lymph node dissection rate, and surgical approach (by means of video-assisted thoracoscopic surgery (VATS), robot-assisted thoracoscopic surgery (RATS), thoracotomy or sternotomy and conversion rate). Tumour characteristics included the number and location of the CRPM. Features of hospital admission included length of stay, complication rate, number of reinterventions, ICU admittance, and readmission rate.

Survival was calculated from the date of metastasectomy to the date of retrieval of survival status or death. Survival for the repeat metastasectomy group was measured from the time of repeat metastasectomy to the date of retrieval of survival status or death. Pulmonary metastasectomy was defined as the first metastasectomy and repeat pulmonary metastasectomy was defined as the second metastasectomy.

The following complications were recorded: persistent air leak (defined as greater than 5 days), bleeding complications, and operative blood loss. Infectious complications include wound infection, empyema, and bronchopleural fistula. Respiratory complications include pneumonia requiring antibiotics, pulmonary oedema, chylothorax, atelectasis requiring bronchoscopy, and phrenic and recurrent nerve injury. Cardiac complications include supraventricular or ventricular arrhythmia, myocardial ischaemia or infarction, and heart failure. Thrombotic complications include deep vein thrombosis, pulmonary embolism, transient ischaemic attack, and cerebrovascular accident. Complications leading to permanent injury were described separately. The composite measure ‘postoperative complicated course’ was defined as a complication leading to prolonged length of hospital stay (greater than 14 days), unplanned reintervention, or mortality rate. This was used to reflect severe complications.

Treatment of bilateral metastases was recorded as simultaneous and staged resections. Staged resections were defined as a second metastasectomy within 3 months and performed on the contralateral side. To demonstrate the national practice and potential variation in the use of metastasectomy for CRPM and non-CRPM between hospitals, the volume of metastasectomy cases for CRPM was analysed as a proportion of the volume of all metastasectomy cases per hospital.

### Statistical analysis

The survival interval was measured from the date of surgery to the date of death from any cause or the date of data extraction from the Vektis database (23 November 2021). Normally distributed continuous variables are presented as medians and 95 per cent c.i., and non-normally distributed data are presented as medians and interquartile ranges (i.q.r.). Categorical variables are presented as frequencies with percentages. Normally distributed continuous data were tested using Student’s *t* test for independent samples. Non-normally distributed data were tested using the Mann–Whitney *U* test. Categorical variables were tested using Pearson’s χ^2^ test or Fisher’s exact test as appropriate. Variables found to be significant in univariable analysis (defined as *P* < 0.100) were included in the multivariable analysis using the Cox proportional hazards model after backward stepwise Wald elimination. Data were tested for normality using the Kolmogorov–Smirnov test, Q–Q plot, and Levene’s test. Missing data were determined to be at random before precluding data from analysis. *P* < 0.050 was considered significant in multivariable analysis. Kaplan–Meier curves were used to compare survival using a log rank test. All analyses were conducted using SPSS^®^ (version 28.0; IBM, Armonk, NY, USA).

## Results

A total of 1337 patients undergoing metastasectomy for CRPM between 2012 and 2019 distributed over 45 hospitals in the Netherlands were registered in the DLCA-S, of whom 1237 (91.8 per cent) were found to be eligible for analysis by meeting the minimal data restrictions and having available survival data. Of these 1237 patients, a total of 127 patients underwent repeat metastasectomy, 17 patients underwent a third metastasectomy, and three patients underwent a fourth metastasectomy.

### Patient characteristics

The mean age of the 1237 patients treated with pulmonary metastasectomy was 65 years and 61.4 per cent were male. Age and sex were not significantly different for repeat metastasectomy (*P* = 0.622). Most patients treated with pulmonary metastasectomy presented with an ECOG performance status of 0 (60.1 per cent). An ASA grade of II or III accounted for 83.9 per cent, and, out of the patients with an available CCI, 84.4 per cent had a CCI of 0 or 1. All three classifications were not significantly different for the repeat metastasectomy group (*P* = 0.194, *P* = 0.538, and *P* = 0.281 respectively). Patient characteristics for both groups are shown in *Table 1*. Nearly all patients (97 per cent) were discussed before surgery by a multidisciplinary team (MDT) tumour board with a median duration of 20 (i.q.r. 13–30) days between the MDT meeting and metastasectomy. The median duration between the first metastasectomy and second metastasectomy was 11 (i.q.r. 6–18) months.

**Table 1:** Characteristics

	Pulmonary metastasectomy (*n* = 1237)	Repeat pulmonary metastasectomy (*n* = 127)
**Age (years), mean(s.d.)**	65(10)	64(10)
**Sex**		
Male	759 (61.4)	87 (68.5)
Female	478 (38.6)	40 (31.5)
**ECOG performance status**		
0	743 (60.1)	76 (59.8)
1	234 (18.9)	28 (22.0)
≥2	27 (2.2)	0 (0)
Unknown/missing	233 (18.8)	23 (18.1)
**ASA classification**		
I	122 (9.9)	9 (7.1)
II	743 (60.1)	81 (63.8)
III	294 (23.8)	29 (22.8)
IV	8 (0.6)	0 (0)
Unknown/missing	70 (5.7)	8 (6.3)
**Charlson co-morbidity index**		
0/1	162 (13.1)	25 (19.7)
2	30 (2.4)	2 (1.6)
Unknown/missing	1046 (84.5)	100 (78.7)
**Pulmonary function test, mean(s.d.)**		
FEV1, % of predicted	99(18)	94(18)
FEV1 <80%	161(13.0)	23(18.1)
FEV1 ≥80%	785(63.5)	72(56.7)
Unknown/missing	291(23.5)	32(25.2)
DLCO, % of predicted, mean(s.d.)	88(18)	82(18)
DLCO <80%	291(23.5)	34(26.8)
DLCO ≥80%	553(44.7)	45(35.4)
Unknown/missing	393(31.8)	48(37.8)
**Cardiopulmonary exercise test**		
VO_2_ max (ml/kg/min), median (i.q.r.)	18.7 (14.9–20.6)	18.3 (16.5–21.5)
Unknown/missing	1195 (96.6)	123 (96.8)

Values are *n* (%) unless otherwise indicated. ECOG, Eastern Cooperative Oncology Group; FEV1, forced expiratory volume in 1 s; DLCO, diffusing capacity of the lungs for carbon monoxide; VO_2_, maximal oxygen consumption; i.q.r., interquartile range.

Mean preoperative FEV1 and DLCO prior to pulmonary metastasectomy were 99 per cent of predicted and 88 per cent respectively and were not different compared with the repeat metastasectomy group (*P* = 0.640 and *P* = 0.569 respectively). Cardiopulmonary exercise testing was available for only 3.4 per cent of patients and was therefore not representative for both groups.

### Survival

**Fig. 1:**
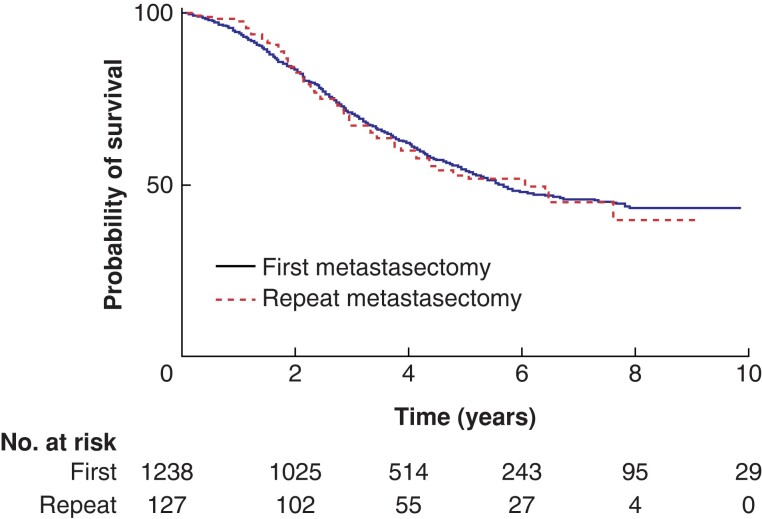
Kaplan–Meier curves of overall survival after first metastasectomy and repeat pulmonary metastasectomy

Overall 90-day mortality rate was 0.6 per cent for patients after pulmonary metastasectomy and 0 per cent for patients after repeat metastasectomy (*P* = 0.364). The median follow-up time for patients was 42 (i.q.r. 28–65; range 0–285) months. During follow-up after repeat metastasectomy, 13.3 per cent and 2.3 per cent of patients underwent a third metastasectomy and a fourth metastasectomy respectively. *Fig. 1* shows the Kaplan–Meier curve for survival for both groups. For pulmonary metastasectomy the 1-, 3-, and 5-year overall survival was 94 per cent, 71 per cent, and 53 per cent respectively. For repeat metastasectomy the 1-, 3-, and 5-year overall survival was 98 per cent, 69 per cent, and 52 per cent respectively. The median survival for pulmonary metastasectomy was 68 (95 per cent c.i. 57 to 79) months and the median survival for repeat metastasectomy was 77 (95 per cent c.i. 51 to 103) months (*P* = 0.852).

### Surgical outcome

The most common type of parenchymal resection was a wedge resection for both first metastasectomy and second metastasectomy (65.0 per cent *versus* 52.8 per cent respectively; *P* = 0.007). Anatomical resections were more frequently performed during pulmonary metastasectomy than repeat metastasectomy (32.7 per cent *versus* 44.6 per cent; *P* = 0.008). Surgical outcomes are shown in *Table 2*. Lymph node dissection rates did not vary between the two groups (14.8 per cent for repeat pulmonary metastasectomy *versus* 14.5 per cent for pulmonary metastasectomy; *P* = 0.393). In the repeat metastasectomy group, 80.5 per cent had a solitary metastasis and 3.1 per cent had four or more metastases resected.

**Table 2:** Surgical outcome

	Pulmonary metastasectomy	Repeat pulmonary metastasectomy	*P*
**Type of surgery**			
Wedge resection	805 (65.0)	67 (52.8)	0.007
Segmentectomy	68 (5.5)	9 (7.1)	0.443
Lobectomy	304 (24.6)	42 (33.1)	0.030
Bilobectomy	15 (1.2)	2 (1.6)	0.717
Pneumonectomy	5 (0.4)	1 (0.8)	0.532
Exploratory thoracotomy	2 (0.2)	0 (0)	0.651
Other	11 (0.9)	2 (1.6)	
**Lymph node dissection**	179 (14.5)	19 (14.8)	0.393
**Surgical approach**			
Minimally invasive	956 (77.2)	82 (64.8)	<0.001
Thoracotomy	256 (20.7)	43 (33.6)	<0.001
Unknown/missing	26 (2.1)	2 (1.6)	
**No. of pulmonary metastases**			
1	1004 (81.2)	103 (80.5)	0.841
2	175 (14.1)	13 (10.2)	0.249
3	28 (2.3)	4 (3.1)	0.496
4	15 (1.2)	4 (3.1)	0.072
Unknown/missing	15 (1.2)	4 (3.1)	
**Postoperative complication**	143 (11.6)	23 (18.1)	0.033
**Postoperative complicated course**	29 (2.3)	7 (5.5)	0.038
**Reintervention**	14 (1.1)	4 (3.1)	0.201
**Permanent injury due to complication**	7 (0.6)	0 (0)	0.633
**Operative blood loss**			
<500 ml	1123 (90.8)	115 (89.8)	0.792
≥500 ml	34 (2.7)	4 (3.2)	
Unknown/missing	80 (6.5)	9 (7.0)	
**Blood transfusion**	7 (0.6)	4 (3.1)	0.018
**Persistent air leak**	39 (3.1)	5 (3.9)	0.231
**Hospital admission**			
Length of stay (days), median (i.q.r.)	4 (3–6)	4 (3–7)	0.639
ICU admittance	179 (14.5)	27 (21.0)	0.332
Readmitted after discharge	16 (1.3)	3 (2.3)	0.046

Values are *n* (%) unless otherwise indicated. i.q.r., interquartile range.

Significantly more patients underwent a minimally invasive resection during the first metastasectomy compared with repeat metastasectomy (77.2 per cent *versus* 64.8 per cent respectively; *P* < 0.001). Only seven metastasectomies were performed by RATS. The conversion rate was 3.2 per cent for the pulmonary metastasectomy group and 9.9 per cent for the repeat metastasectomy group (*P* < 0.001). The conversion was 16.1 per cent if the previous metastasectomy was performed on the same side. The pulmonary metastasectomy group contained 28 strategic conversions and four reactive conversions. The repeat metastasectomy group contained eight strategic conversions and one reactive conversion.

Out of the 127 repeat metastasectomy procedures, 46 patients (36.2 per cent) had a previous metastasectomy on the contralateral side, 65 patients (51.2 per cent) had a previous VATS metastasectomy on the same side, and 16 patients (12.6 per cent) had a previous open metastasectomy on the same side. In the pulmonary metastasectomy group, 61 patients underwent a bilateral procedure. This included a staged resection in 46 patients and simultaneous resection in 17 patients. All repeat metastasectomy cases were unilateral resections.

The length of hospital stay was not different for the two groups with a median duration of 4 days (*P* = 0.639). Significantly more patients experienced postoperative complications due to repeat metastasectomy (18.1 per cent *versus* 11.6 per cent; *P* = 0.033). If a previous metastasectomy was performed on the same side, postoperative complications were higher than in patients with a previous metastasectomy on the other side (23.8 per cent *versus* 8.7 per cent respectively; *P* = 0.027). No significant difference in complication rate was observed between a previous open metastasectomy and a previous VATS metastasectomy (31.3 per cent *versus* 21.9 per cent; *P* = 0.311). More patients experienced a postoperative complicated course for repeat metastasectomy (5.5 per cent *versus* 2.3 per cent; *P* = 0.038). In the pulmonary metastasectomy group, 5.2 per cent experienced respiratory complications, 1.0 per cent experienced infectious complications, 1.1 per cent experienced cardiac complications, and 0.7 per cent experienced thrombotic complications. In the repeat metastasectomy group, 8.6 per cent experienced respiratory complications, 3.9 per cent experienced infectious complications, 2.3 per cent experienced cardiac complications, and 0.8 per cent experienced thrombotic complications. No difference in persistent air leak (3.9 per cent *versus* 3.1 per cent), complications leading to permanent injury (0.0 per cent *versus* 0.6 per cent), reintervention rate (3.1 per cent *versus* 1.1 per cent), and ICU admittance (21.0 per cent *versus* 14.5 per cent) was noted between the two groups (repeat pulmonary metastasectomy and pulmonary metastasectomy respectively). No difference was observed for excessive operative blood loss (3.2 per cent *versus* 2.7 per cent for repeat pulmonary metastasectomy and pulmonary metastasectomy respectively). However, more patients required a blood transfusion after repeat metastasectomy (3.1 per cent *versus* 1.8 per cent).

Non-anatomical (wedge) resections were more frequently performed during VATS procedures than open procedures, with 73.4 per cent compared with 41.9 per cent respectively (*P* < 0.001). Lymph node dissection was more frequently performed during open procedures than VATS procedures, with 25.9 per cent compared with 13.2 per cent respectively (*P* < 0.001). However, lymph node dissection rates were similar for multiple metastases compared with solitary metastases, with 15.6 per cent compared with 11.5 per cent (*P* = 0.082). Postoperative complications were noted more frequently for open procedures than VATS procedures, with 23.6 per cent compared with 9.2 per cent respectively (*P* < 0.001). Complications were also noted more frequently after anatomical resections than non-anatomical resections, with 20.8 per cent compared with 8.0 per cent respectively (*P* < 0.001).

### Prognostic factors for survival

*Table 3* and *Table 4* document the univariable and multivariable Cox regression for both groups. On univariable analysis for pulmonary metastasectomy, four prognostic factors for survival were identified: age (hazards ratio (HR) 1.01, 95 per cent c.i. 1.00 to 1.02; *P* = 0.050), ECOG performance status greater than or equal to 1 (HR 1.34, 95 per cent c.i. 1.09 to 1.66; *P* = 0.006), multiple metastases (HR 1.35, 95 per cent c.i. 1.10 to 1.66; *P* = 0.004), and bilateral metastases (HR 1.57, 95 per cent c.i. 1.12 to 2.19; *P* = 0.008). On multivariable analysis, ECOG performance status greater than or equal to 1 (HR 1.33, 95 per cent c.i. 1.08 to 1.65; *P* = 0.008), multiple metastases (HR 1.30, 95 per cent c.i. 1.01 to 1.67; *P* = 0.038), and bilateral metastases (HR 1.50, 95 per cent c.i. 1.01 to 2.22; *P* = 0.045) remained significant prognosticators.

**Table 3:** Prognostic factors for overall survival after pulmonary metastasectomy for colorectal pulmonary metastases

	Univariable analysis		Multivariable analysis	
	HR (95% c.i.)	*P*	HR (95% c.i.)	*P*
**Sex**				
Male	1.10 (0.92,1.31)	0.304		
Female	1.00			
**Age (years)**	1.01 (1.00,1.02)	0.050	1.01 (1.00,1.02)	0.084
**ECOG**				
0	1.00			
≥1	1.34 (1.09,1.66)	0.006	1.33 (1.08,1.65)	0.008
**ASA**				
I	1.00			
II	1.00 (0.76,1.32)	0.991		
III	1.11 (0.81,1.51)	0.512		
IV	1.95 (0.71,5.37)	0.201		
**Pulmonary function**				
FEV1 <80%	1.00 (0.99,1.00)	0.418		
DLCO <80%	1.00 (0.99,1.00)	0.485		
VO_2_ max	0.92 (0.84,1.01)	0.084		
**No. of metastases**				
Solitary	1.00			
Multiple	1.35 (1.10,1.66)	0.004	1.30 (1.01,1.67)	0.038
**Location of metastases**				
Unilateral	1.00			
Bilateral	1.57 (1.12,2.19)	0.008	1.50 (1.01,2.22)	0.045
**Type of resection**				
Non-anatomical	1.00			
Anatomical	1.17 (0.95,1.36)	0.170		
**Lymph node dissection**	1.00 (0.78,1.29)	0.983		
**Complications**	1.14 (0.88,1.48)	0.311		

HR, hazards ratio; ECOG, Eastern Cooperative Oncology Group; FEV1, forced expiratory volume in 1 s; DLCO, diffusing capacity of the lungs for carbon monoxide; VO_2_, maximal oxygen consumption.

**Table 4:** Prognostic factors for overall survival after repeat metastasectomy for colorectal pulmonary metastases

	Univariable analysis		Multivariable analysis	
	HR (95% c.i.)	*P*	HR (95% c.i.)	*P*
**Sex**				
Male	1.80 (0.97,3.35)	0.062	0.84 (0.35,2.00)	0.686
Female	1.00			
**Age (years)**	1.02 (0.99,1.05)	0.185		
**ECOG**				
0	1.00			
≥1	1.40 (0.74,2.64)	0.297		
**ASA**				
I	1.00			
II	1.78 (0.55,5.80)	0.336		
III	1.82 (0.51,6.46)	0.357		
**Pulmonary function**				
FEV1 <80%	1.00 (0.99,1.02)	0.675		
DLCO <80%	1.03 (1.01,1.05)	0.009	1.04 (1.01,1.06)	0.004
VO_2_ max	1.92 (0.45,8.24)	0.381		
**No. of metastases**				
Solitary	1.00			
Multiple	1.87 (0.98,3.58)	0.058	2.04 (0.82,5.05)	0.123
**Type of resection**				
Non-anatomical	1.00			
Anatomical	1.46 (0.84,2.54)	0.183		
**Lymph node dissection**	0.61 (0.24,1.53)	0.289		
**Complications**	0.47 (0.19,1.17)	0.104	0.47 (0.16,1.40)	0.175

HR, hazards ratio; ECOG, Eastern Cooperative Oncology Group; FEV1, forced expiratory volume in 1 s; DLCO, diffusing capacity of the lungs for carbon monoxide; VO_2_, maximal oxygen consumption.

On univariable analysis for repeat metastasectomy, four prognostic factors for survival were identified: male sex (HR 1.80, 95 per cent c.i. 0.97 to 3.35; *P* = 0.062), DLCO less than 80 per cent (HR 1.03, 95 per cent c.i. 1.01 to 1.05; *P* = 0.009), multiple metastases (HR 1.87, 95 per cent c.i. 0.98 to 3.58; *P* = 0.058), and postoperative complications (HR 0.47, 95 per cent c.i. 0.19 to 1.17; *P* = 0.101). On multivariable analysis, only DLCO less than 80 per cent (HR 1.04, 95 per cent c.i. 1.01 to 1.06; *P* = 0.004) remained a significant prognostic factor.

**Fig. 2:**
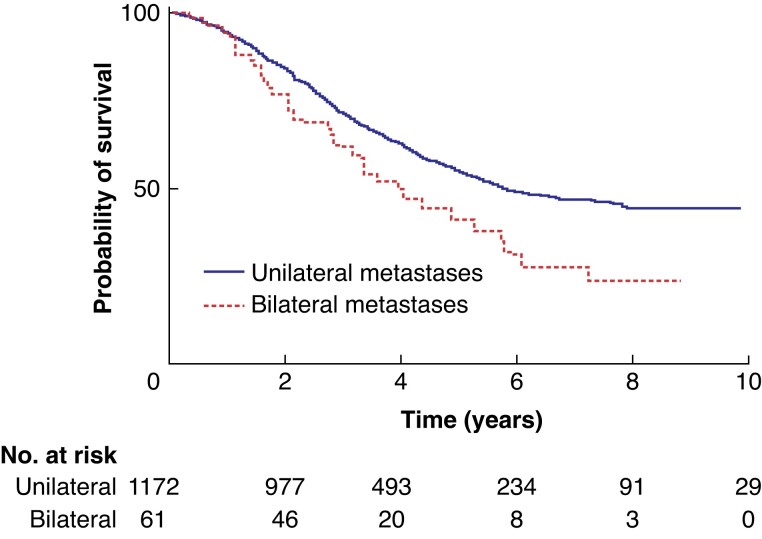
Kaplan–Meier curves of overall survival after metastasectomy for unilateral metastases and bilateral metastases

**Fig. 3:**
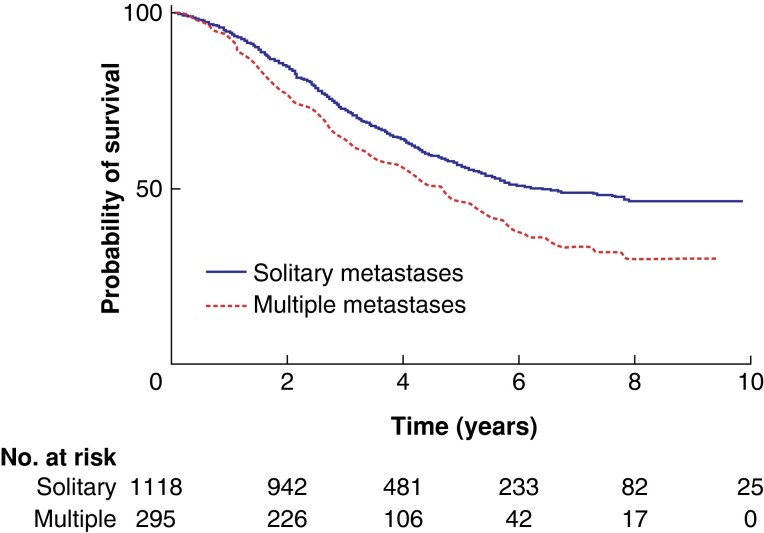
Kaplan–Meier curves of overall survival after metastasectomy for solitary metastases and multiple metastases

*Fig. 2* demonstrates the 5-year overall survival after metastasectomy of unilateral metastases (54 per cent) and bilateral metastases (40 per cent) (*P* = 0.008). *Fig. 3* shows the 5-year overall survival rate for metastasectomy of solitary metastases (55 per cent) and multiple metastases (45 per cent) (*P* < 0.001).

### Volume of metastasectomy cases

**Fig. 4:**
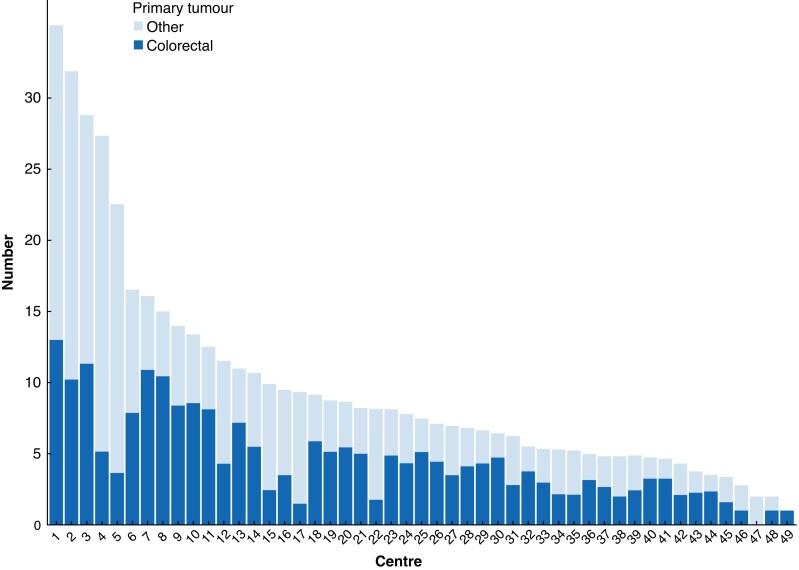
Mean volume of pulmonary metastasectomy cases for non-colorectal and colorectal metastases per individual hospital in the Netherlands

As shown in *Fig. 4*, there was a wide variation between hospitals with a thoracic surgery unit in the volume of metastasectomy cases for both CRPM and non-CRPM. A mean of 169 patients underwent a metastasectomy per year for CRPM, increasing to 194 patients per year when including sequential metastasectomy cases. An almost yearly increase in the number of pulmonary metastasectomy cases for CRPM can be seen, with 122 cases in 2012, 149 cases in 2015, and 182 cases in 2019. During the study interval, the rate of VATS resections for CRPM significantly increased over time, with 64.6 per cent of procedures in 2012, 76.5 per cent of procedures in 2015, and 90.1 per cent of procedures in 2019 (*P* < 0.001). The median number of metastasectomy cases per hospital per year for CRPM was 3.9 (i.q.r. 2.4–5.5, range 1–11.5) and the median number of metastasectomy cases per hospital per year for all pulmonary metastases was 6.5 (i.q.r. 4.2–10.1, range 1–34.5).

### Third metastasectomy and fourth metastasectomy

Seventeen patients underwent a third metastasectomy during follow-up. The median interval between repeat metastasectomy and third metastasectomy was 9 (i.q.r. 5–26) months. The 1-, 3-, and 5-year overall survival was 94 per cent, 88 per cent, and 63 per cent respectively. Three patients also underwent a fourth metastasectomy with a median interval between the third metastasectomy and fourth metastasectomy of 8 (i.q.r. 3–12) months.

## Discussion

The results of the current study demonstrate similar 5-year overall survival when comparing the first pulmonary metastasectomy with repeat metastasectomy for CRPM (53 per cent *versus* 52 per cent respectively) in selected patients. The median survival for pulmonary metastasectomy was 68 (95 per cent c.i. 57–79) months and the median survival for repeat metastasectomy was 77 (95 per cent c.i. 51–103) months (*P* = 0.852). These results suggest that repeat metastasectomy leads to good long-term survival rates in selected patients with colorectal cancer. Other reports on repeat metastasectomy have provided similar survival rates^13,14,16,17^. Several retrospective studies have attempted to compare patients’ survival rates after repeat metastasectomy to patients without metastasectomy for lung-limited recurrence^2,9,10^. However, the selection of patients towards a specific treatment option is a determining factor for the outcome of a patient^27^. These results do not demonstrate that additional survival can be attributed to the effect of metastasectomy.

Studies on repeat metastasectomy of CRPM have reported variable 5-year overall survival rates of 51–79 per cent^9-18^. However, these results must be interpreted carefully. Care should be taken not to include the time between the first metastasectomy and second metastasectomy when analysing repeat metastasectomy survival rates. Furthermore, some studies have analysed pulmonary metastases of all tumour types and performed regression techniques to provide evidence on colorectal histology^11,12,15^. Some studies are single-institution studies with a small sample size limiting external validity and power^2,14,17,19^. A total of 127 patients that underwent repeat pulmonary metastasectomy were recorded in the DLCA-S database (10 per cent of all metastasectomy cases). The rates of repeat pulmonary metastasectomy vary from 10 to 26 per cent^2,9-11,14,16-19^. The median time interval between first resection and second resection was 12 months. Other reviews have demonstrated variable interval times of 13–19 months^4,5,7,9,10,14,17^.

To better understand the selection process that occurs in patients with colorectal metastases, the number of patients receiving local treatment for both colorectal lung and liver metastases in the Netherlands were compared. Annually, 10 779 patients undergo surgery for colorectal cancer^28^, 194 patients undergo metastasectomy for pulmonary metastases^29^, and 955 patients undergo resection of colorectal liver metastases^30^. Therefore, 1.8 per cent of patients after resection for colorectal cancer will undergo pulmonary metastasectomy and 8.9 per cent of patients will undergo surgical resection for colorectal liver metastases. However, based on previous Dutch reports, 12–15 per cent of patients present with metachronous isolated lung metastases and 31 per cent present with metachronous isolated colorectal liver metastases^30,31^.

In the repeat metastasectomy group, the rate of postoperative complications was higher. The difference in postoperative complications was attributed to relatively more respiratory and infectious complications. However, the median length of stay, reintervention rate, and 90-day mortality rate were not different for the two groups. Review of the repeat metastasectomy group revealed that this difference was mainly attributed to the group of patients that underwent a previous metastasectomy on the same side. A tendency was noted that a previous open metastasectomy resulted in a higher incidence of postoperative complications than a previous VATS metastasectomy. Other studies on repeat metastasectomy have not demonstrated a difference in postoperative morbidity rate after repeat metastasectomy compared with the first metastasectomy^12,13^. A study analysing 46 patients after repeat metastasectomy demonstrated that a previous VATS procedure was associated with fewer pleural adhesions and shorter operating times during repeat metastasectomy than a previous thoracotomy^32^. The increased rate of postoperative complications in the repeat metastasectomy group can in part be explained by the higher rate of open procedures and anatomical resections in this group. The results from the DLCA-S database are in line with the European Society of Thoracic Surgeons (ESTS) database that revealed postoperative complication rates of 15 per cent^33^.

Several well-documented parameters are considered unfavourable prognostic factors for pulmonary metastasectomy, such as short disease-free interval, number of metastases, elevated carcinoembryonic antigen (CEA), previous liver metastasis, positive surgical margins, and involved hilar or mediastinal lymph nodes^1,23,24^. Multivariable regression analysis of the DLCA-S database revealed ECOG performance status, multiple metastases, and bilateral metastases as significant prognostic variables for the first metastasectomy. For repeat metastasectomy, these variables were not significant prognosticators. It is questionable whether impaired pulmonary function tests should be considered a negative prognosticator and there were high rates of missing data on pulmonary function. Other studies have demonstrated by multivariable analyses that histological grade of the primary tumour^9^, size of the metastases^16^, CEA concentration^14^, and history of colorectal liver metastases^10^ are prognosticators for patients after repeat metastasectomy. However, most reports present limited to no prognostic factors for repeat metastasectomy^9-18^.

Recent analysis of the ESTS database demonstrated a yearly increase in the percentage of metastasectomy cases being performed by VATS, with 30 per cent in 2012 and 59 per cent in 2019^33^. The DLCA-S registry demonstrates the same tendency towards minimally invasive metastasectomy with 65 per cent in 2012 and 90 per cent in 2019. Both the ESTS database and the Dutch database reveal a low conversion rate (2.1 and 3.2 per cent respectively). The conversion rate for repeat metastasectomy was 10 per cent, and 16 per cent if the previous metastasectomy was performed on the same side. In the last decade, minimally invasive approaches for pulmonary metastases have gained acceptance and radiological imaging has considerably improved, facilitating the transition towards minimally invasive metastasectomy.

A relatively low percentage of patients (15 per cent) underwent lymph node dissection during metastasectomy. Meanwhile, lymph node dissection was more frequently performed during open procedures (26 per cent). The ESTS database^33^ revealed that lymph node dissection was performed in 57 per cent and noted that 70 per cent of patients underwent lymph node assessment during open procedures. Both studies show that surgeons are more likely to perform lymph node dissection during an open metastasectomy. There is no evidence that lymph node dissection improves survival. The incidence of lymph node metastases during metastasectomy for CRPM is 19 per cent^24^. Hilar or mediastinal lymph node metastases are considered a prognostic factor (HR 1.65, 95 per cent c.i. 1.35 to 2.02), and mediastinal lymph node metastases have an estimated 5-year overall survival rate of 10.9 per cent after metastasectomy^1,24^. It remains questionable whether the added prognostic value of performing lymph node dissection outweighs the added morbidity rate. A Danish randomized clinical trial is currently assessing the impact of pulmonary metastasectomy with lymphadenectomy on CRPM^34^.

Limitations of this study include the lack of data on other local treatments (by means of stereotactic radiotherapy or percutaneous ablation) as well as the systemic treatment of registered patients. Furthermore, no information is presented regarding the treatment of other colorectal metastases. These data can severely affect survival rates. Preoperative imaging characteristics have not been included in the DLCA-S registry as well as the treatment of the primary tumour. Therefore, it was not possible to use all known prognostic factors for multivariable analyses. Hopefully, the ongoing evolution of data entry will allow for a better insight into current practice for colorectal cancer patients with oligometastatic and lung-limited disease. More importantly, to assess the true value of (repeat) metastasectomy for colorectal cancer, more data should become available on patients receiving other forms of local and systemic therapy. The Dutch Lung Cancer Audit will have to continuously evolve to better analyse patients and the treatment for CRPM.

## Data Availability

Raw data were generated by Medical Research Data Management (MRDM). Derived data supporting the findings of this study are available from the corresponding author (M.v.D.) on request.
